# The Underestimated Significance of Conditioning in Placebo Hypoalgesia and Nocebo Hyperalgesia

**DOI:** 10.1155/2018/6841985

**Published:** 2018-01-28

**Authors:** Anne-Kathrin Bräscher, Michael Witthöft, Susanne Becker

**Affiliations:** ^1^Department for Clinical Psychology, Psychotherapy, and Experimental Psychopathology, Johannes Gutenberg University Mainz, Mainz, Germany; ^2^Department of Cognitive and Clinical Neuroscience, Central Institute of Mental Health, Medical Faculty Mannheim, Heidelberg University, Heidelberg, Germany

## Abstract

Placebo and nocebo effects are intriguing phenomena in pain perception with important implications for clinical research and practice because they can alleviate or increase pain. According to current theoretical accounts, these effects can be shaped by verbal suggestions, social observational learning, and classical conditioning and are necessarily mediated by explicit expectation. In this review, we focus on the contribution of conditioning in the induction of placebo hypoalgesia and nocebo hyperalgesia and present accumulating evidence that conditioning independent from explicit expectation can cause these effects. Especially studies using subliminal stimulus presentation and implicit conditioning (i.e., without contingency awareness) that bypass the development of explicit expectation suggest that conditioning without explicit expectation can lead to placebo and nocebo effects in pain perception. Because only few studies have investigated clinical samples, the picture seems less clear when it comes to patient populations with chronic pain. However, conditioning appears to be a promising means to optimize treatment. In order to get a better insight into the mechanisms of placebo and nocebo effects in pain and the possible benefits of conditioning compared to explicit expectation, future studies should carefully distinguish both methods of induction.

## 1. Introduction

Placebo and nocebo effects are prevalent topics in current research, especially in the domain of pain, where they can be investigated comparatively easily and serve as a model for other systems (e.g., immune, motor, and respiratory systems [1]). While placebo hypoalgesia refers to decreased pain sensitivity due to an inert treatment (e.g., sham procedure and inert substance), its counterpart nocebo hyperalgesia is defined as increased pain sensitivity attributable to an inert treatment [2]. Due to their capacity to improve or worsen symptoms and well-being, placebo and nocebo effects are highly relevant not only in research but also in clinical practice. They contribute to most therapeutic effects [3], occur regularly in doctor-patient interactions [4], and are assumed to play a role in the development and maintenance of chronic pain and other diseases [5, 6]. Placebo and nocebo effects also significantly influence the outcome of randomized placebo-controlled clinical trials, for example, leading to an underestimation of clinical effects of novel drugs [7–9]. Although this clinical relevance and research on placebo and nocebo effects have prospered during the past decade, many issues remain unresolved or poorly understood. One of these issues that deserve our attention concerns the causing mechanisms of placebo and nocebo effects and the significance of conditioning in this realm. Only if we understand how these effects emerge, we can systematically take advantage of placebo and avoid nocebo effects, especially in the clinical context.

After giving a brief overview over current evidence and models on the development of placebo hypoalgesia and nocebo hyperalgesia including conditioning and expectation approaches, we will turn to studies that stress the significance of conditioning and demonstrate that conditioning effects might have been misunderstood and/or underestimated. We will highlight the need to better understand conditioned placebo and nocebo effects and propose experimental designs that allow a conclusive investigation of the specific mechanisms at work.

## 2. Development of Placebo and Nocebo Effects

Placebo and nocebo effects can be induced by different means. Empirical evidence shows that verbal suggestion [10], classical conditioning [11–13], and observational learning [14, 15] can lead to placebo hypoalgesia and nocebo hyperalgesia. Verbal suggestions in terms of written or spoken statements on the alleged effect of the placebo or nocebo are thought to induce an explicit expectation that directly mediates the effect [16, 17]. In contrast, direct experience of a pain-increasing or -relieving effect is essential in conditioning of placebo hypoalgesia and nocebo hyperalgesia [18]. Here, a placebo/nocebo serves as conditioned stimulus and is repeatedly coupled to a pain stimulus (unconditioned stimulus), in which the intensity is reduced or increased without the person's knowledge compared to before placebo/nocebo administration or to a simultaneous stimulation without placebo/nocebo. Subsequently, the conditioned effect is tested by retracting the surreptitious change in stimulation intensity while still administering the placebo/nocebo. Observational learning of placebo and nocebo effects has been investigated only recently. It is usually carried out by showing videos of or live models who are demonstrating reduced or increased pain sensations upon administration of a placebo/nocebo [15, 19–21]. It is assumed that the observation serves as an unconditioned stimulus [22].

Typically, conditioning and verbal suggestions are both implemented in experimental studies to increase placebo/nocebo effects in pain [23–26]. Accordingly, meta-analyses come to the conclusion that the combination of conditioning and verbal suggestion leads to larger effects than verbal suggestion alone [27, 28]. When inducing placebo hypoalgesia or nocebo hyperalgesia by verbal suggestions alone, results are inconsistent, sometimes resulting only in weak [24] or even no effects [25, 29, 30] and at other times leading to robust pain reduction [10, 31] or increase [10, 32]. Some studies indicate that learning is less important in nocebo hyperalgesia compared to placebo hypoalgesia, but direct comparisons are rare [24, 33]. Interestingly, while placebo effects induced by expectation can be antagonized by the opioid antagonist naloxone, conditioned placebo effects can be antagonized by naloxone when the pharmacological conditioning had been performed with an opioid and by cannabinoid receptor antagonists when it had been performed with ketorolac, a nonsteroidal anti-inflammatory drug [34, 35]. These findings highlight that placebo and nocebo effects in pain differ depending on whether they were induced by verbal suggestion or conditioning.

In a series of three studies, Benedetti et al. [10] showed that (a) placebo and nocebo effects in hormone secretion (growth hormone and cortisol) were affected by pharmacological conditioning, but not by verbal suggestions, (b) placebo and nocebo effects in pain were induced by pharmacological conditioning and by verbal suggestions, but pharmacologically conditioned placebo hypoalgesia was overridden by opposing verbal suggestions, and (c) motor performance in Parkinsonian patients depended on verbal suggestion after repeated deactivation of implanted stimulating electrodes. Based on these observations, the authors developed an influential model stating that conditioning can directly lead to placebo and nocebo effects in unconscious processes (e.g., hormone secretion) and that expectation can lead to placebo and nocebo effects in conscious processes (e.g., pain and motor performance) and that effects of conditioning on conscious processes are necessarily mediated by explicit expectation. Furthermore, it is assumed that expectation cannot directly affect unconscious processes [10]. Although these conclusions fit to the results of the presented studies, we assert that the model underestimates conditioned effects, as outlined below.

Previous research on the mechanisms of placebo and nocebo effects might be biased due to a number of reasons: (1) studies that investigate the effects of conditioning without verbal suggestion are scarce [30, 36, 37]. In order to estimate the contribution and investigate the mechanisms of conditioning to a given effect, it is likely not as simple as to subtract the effect size from a verbal suggestion group from that of a verbal suggestion plus conditioning group. It could well be that conditioning and verbal suggestion do not interact in an additive manner, although this seems to be assumed in most studies. (2) When investigating the effects of a conditioning procedure without verbal suggestions, oftentimes a medically connoted placebo or nocebo (e.g., ointment, pill, and sham acupuncture) is used [13, 29, 38, 39]. However, these placebos and nocebos most probably induce expectations from the outset independent of the experimental manipulation. Participants might have been preconditioned by previous experiences with similar medical devices and procedures. As a consequence, unintended additional expectations will develop that complicate disentangling expectation- and conditioning-induced effects. Evidence supporting this assumption comes from a study by Montgomery and Kirsch [39], in which a small placebo effect emerged after applying an inert tincture without giving a verbal suggestion and before conditioning. (3) Another pitfall of many studies testing conditioned effects is the implementation of the conditioning procedure itself. Due to credibility, a medically connoted placebo or nocebo, like an ointment, will only be applied once in the beginning of the study, for example, on the right arm, and then tested against a spot without ointment, for example, on the left arm [12, 13, 39–42]. In other cases, the placebo/nocebo is applied only very few times [11, 43]. Proper conditioning, however, relies on repeated pairings of the conditioned stimulus (i.e., the placebo or nocebo) and the unconditioned stimulus (i.e., reduced or increased pain) (e.g., [44]). It is known from the conditioning literature that conditioned effects are stronger when more pairings of CS and US are applied [45]. By applying a placebo/nocebo only once, it cannot be ensured that the pairing is processed as intended. To summarize, most previous studies do not allow disentangling of placebo/nocebo effects induced by conditioning and/or explicit expectations or do not use adequate conditioning procedures to test for conditioned effects so that conditioned placebo and nocebo effects can hardly be evaluated.

## 3. Mediation by Expectation Hypothesis

An important aspect of the above-cited model [10] concerns the necessary mediation of conditioning effects in pain via explicit expectation. Colloca and Miller [22] developed a learning perspective on placebo responses by applying Peirce's theory of signs and refining Benedetti's model [10]. In addition to Benedetti's model, they state that signs in the form of indices (i.e., conditioned stimuli), symbols (i.e., communication), and icons (i.e., observations) are detected and processed, resulting in the formation of expectations, which thereby contribute to placebo and nocebo responses. In line with Benedetti's model, bodily functions that are consciously accessible, as pain relief, are mediated by expectation, but “an event that cannot be experienced and perceived by human cognition (e.g., growth factor secretion) appears not to be influenced by self-cognition” (p. 1865). Although the authors endorse the possibility of conditioning without awareness, and accordingly unconscious expectations, they argue that it is circumstantial in creatures with higher phylogenetic level. In the following, we will show that conditioned placebo hypoalgesia and nocebo hyperalgesia are not necessarily mediated by explicit expectations, emphasizing the assumption that the conditioned effect can exist independent of explicit expectation.

Many studies report correlations between pain and expected pain ratings [17, 39, 46, 47], sometimes on a trial-by-trial basis [26], after conducting a conditioning procedure. Using a mediation analysis, it has been shown that conditioning is highly related to expectancy and expectancy predicts the placebo effect, while the direct effect of conditioning on the placebo effect is no longer significant [38]. This result suggests that conditioning is mediated by expectation. However, the robustness of this outcome seems somewhat uncertain, as a bias-corrected bootstrap approach was not significant. A study of Montgomery and Kirsch [39] can serve as another example for mediation by expectation. They compared a conditioned group (uninformed pairing) to a group with informed pairing (i.e., participants underwent a conditioning procedure but were informed about the intensity reduction on placebo trials), and a control group that was not conditioned. The strongest placebo effect appeared in the uninformed pairing group, but when including expectancy as a covariate, group differences disappeared and expectancy was significantly related to placebo effects. Furthermore, some studies report that conditioned effects can be blocked by opposite verbal suggestions [10, 39]. However, this is not always the case, as there are studies showing conditioned effects despite opposite verbal suggestions [11, 12] or expectancy [48]. Also, conditioned effects have been shown to be mediated by expectancy only partially [49], and expectation ratings do not necessarily predict conditioned effects [37, 50] or correspond to pain ratings [29]. In a study of De Pascalis et al. [41], two placebo creams were administered with supposedly “strong” and “weaker” dosage. Subsequently, only after application of the “strong placebo,” the intensity of electric pain stimuli was surreptitiously reduced. Although expected pain level varied according to the manipulation (i.e., strong placebo led to higher expectation of pain relief compared to weak placebo), pain ratings did no differ after strong and weak placebo, highlighting the dissonance between expectations and measured placebo hypoalgesia.

Studies on open-label placebo administration give another important insight into the mediating role of expectation in placebo hypoalgesia. Typically, open-label placebo studies try to boost expectation of a positive effect despite openly administering an inert substance [51, 52]. However, a highly intriguing study in healthy participants showed that conditioned placebo hypoalgesia persisted after revealing the inert nature of a placebo intervention (cream) independent of the participants' expectation. Other than in the previously mentioned open-label placebo studies, participants in this study were not encouraged to believe in a positive effect from the placebo cream. Although participants no longer expected a hypoalgesic effect, the placebo effect remained [50]. In a recent systematic review and meta-analysis on open-label placebo studies, it was hypothesized that the mechanism driving such effects is classical conditioning [53].

In an editorial, Wager [54] begs interesting questions on the role of expectation as a mediator of placebo effects. He argues that, despite the predictive value of expectation for placebo effects, a causal relationship is not necessarily implied. Besides a direct influence of expectation on pain experience, he proposes three alternative explanations for the observed effects: expectancy could affect reported pain ratings (1) directly, (2) via a kind of social contract that is initiated after giving an expectancy rating, and (3) as a consequence of the third variable that affects both expectancy and pain rating, like demand characteristics, personality traits, treatment history, or situational factors. Supporting these ideas, a study of de Jong et al. [29] can be consulted, in which three different groups were investigated: the experimental group underwent a conditioning procedure with additional verbal suggestions, control group 1 underwent the same conditioning procedure but was told that stimulus intensities would be halved during trials, in which the placebo was applied, and that an inert substance was used, and control group 2 received verbal suggestions only. Despite similar differential expectations of the experimental group and control group 2, only the experimental group showed placebo hypoalgesia. Although no placebo effect was found in control group 1, pain ratings did not differ between the experimental group and control group 1, suggesting a relative independence from verbally induced expectations. Yet, expectation was found to correlate with the placebo hypoalgesia, however, irrespective of the experimental manipulation. The authors speculate that this correlation reflects an interaction between individual traits and general characteristics of the placebo used, thus possibly serving as an example for Wager's third case.

In conclusion, mediation by expectation of conditioned placebo hypoalgesia and nocebo hyperalgesia can occur but in many cases does not. We therefore now turn to studies that demonstrate the significance of conditioning effects independent of explicit expectation.

## 4. Evidence for the Significance of Classical Conditioning

Evidence of different research areas highlights the significance of conditioning of placebo hypoalgesia and nocebo hyperalgesia that is independent of explicit expectation. It has been shown that besides humans, animals, such as rodents, can develop conditioned placebo hypoalgesia and nocebo hyperalgesia [55–57]. Furthermore, a limited number of studies implemented placebo or nocebo conditioning procedures without additional verbal suggestions and used meaningless cues as conditioned stimuli (e.g., red and green lights), instead of medically connoted substances or procedures, bypassing some of the abovementioned limitations of previous studies. Results are inconsistent: while recent studies found placebo hypoalgesia and nocebo hyperalgesia after conditioning without additional verbal suggestions that were not predicted by expectancy ratings [37], or without contingency awareness [58], other studies reported no placebo hypoalgesia or nocebo hyperalgesia after conditioning without additional verbal suggestions [30, 36].

A line of research that strongly supports the existence of conditioned placebo and nocebo effects in pain that are independent of explicit expectation uses subliminal cues and implicit conditioning, that is, conditioning without contingency awareness. Some studies showed that placebo hypoalgesia and nocebo hyperalgesia that had been conditioned with supraliminally presented cues (i.e., explicit conditioning) can be activated by subliminally presented cues [21, 59]. Jensen et al. [60], for instance, conditioned healthy participants with the display of faces and activated placebo hypoalgesia and nocebo hyperalgesia by supraliminal as well as subliminal face presentation [60]. These results were replicated in a functional magnetic resonance imaging (fMRI) study of the same working group [61].

One attempt to implicitly condition placebo hypoalgesia by using a tactile cue (direction in which a placebo cream was applied to the skin) was not successful in inducing placebo hypoalgesia [43], but a recent study indicated that contingency awareness is not necessary to induce a nocebo effect in heat-pain perception [58]. So far, there is only one study that used a subliminal stimulus presentation also during the acquisition phase of a conditioning experiment in order to rule out explicit expectation in conditioning [44]. The authors assigned healthy participants to one of four groups, each including an acquisition phase and a test phase and varying subliminal and supraliminal stimulus presentations. Overall, F tests indicated that there was no difference in placebo hypoalgesia and nocebo hyperalgesia between the different types (i.e., subliminal/supraliminal) of the acquisition or test phase, strongly suggesting the presence of implicitly conditioned effects.

In order to gain more insight into the role of conditioned effects on placebo and nocebo effects in pain, research on implicit conditioning should be expanded and diversified, as previous studies mostly did not directly test for contingency awareness and typically focused on subliminal conditioning, which has some pitfalls (e.g., intra- and interindividually varying threshold for subliminal stimulus presentation). Although accumulating evidence points to an important role of conditioning of placebo and nocebo effects in pain independent of explicit expectations, most current theoretical accounts do not yet incorporate these aspects sufficiently [10, 22]. One point of criticism regarding prevalent models concerns the classification of physiological processes into being either conscious or unconscious. Evidence suggests that it is conceivable that most, if not all, bodily functions, including perception and behavior, have “unconscious” (i.e., outside a person's awareness; cf., e.g., blindsight and implicit operant conditioning [62–64]) and “conscious” (i.e., verbally represented) portions. Pain, especially, is known as a multidimensional phenomenon that incorporates sensory, affective, behavioral, and physiological levels that can all be accessed on aware and unaware levels [63–66]. An exception provides a theoretical framework of Haug [67], which, however, has not received much attention. He proposes that placebo/nocebo effects are mediated by so-called aliefs, which are subdoxastic states, that is, cognitive states “with consciously inaccessible content which is inferentially isolated from the subject's large network of beliefs (and which may directly cause behavior)” (p. 690). Aliefs can be consciously or unconsciously activated in humans and nonhumans by the internal or external environment. They are associative, automatic, arational, affect-laden, and action generating and might be a better candidate for a common final path than expectation in Colloca and Miller's [22] model because they can explain placebo and nocebo effects that have been induced by verbal suggestion as well as (implicit) conditioning.

## 5. Clinical Context

While the majority of studies on placebo hypoalgesia and nocebo hyperalgesia examined healthy participants, only few investigated pain patients and even less focused on clinical pain [68]. Nonetheless, the available studies give some insights into the mechanisms of placebo and nocebo effects in a clinical pain context.

Verbal suggestion of pain relief seems to be highly effective for acute procedural (large effect with Hedges' *g* = 1.03) as well as chronic pain (small effect with Hedges' *g* = 0.25 [68, 69]) even after open-label placebo administration in patients with irritable bowel syndrome (IBS [51]) and chronic low back pain [52]. Furthermore, large placebo effects (comparable to the effect of the local anesthetic Lidocain) have been induced by using verbal suggestions and application of a placebo (lubricant) during the induction of experimental pain (rectal distention) in patients with IBS [70, 71]. While explicit expectations accounted for large amounts of the variance in experimental visceral pain during placebo and Lidocain administration, it was not predictive for a cutaneous pain model despite placebo hypoalgesia being present [70]. In a similar study, expectation did predict the placebo and Lidocain effect in the late phase of experimental visceral pain, but not in the early phase [71].

Placebo effects have also been shown in studies that combined verbal suggestion and conditioning in patients with IBS [72], knee osteoarthritis [73], atopic dermatitis [40], musculoskeletal pain [74], and chronic low back pain [69]. Results of a meta-analysis show that, in pain patients, medium-sized placebo hypoalgesia (Hedges' *g* = 0.65) was induced with a combination of verbal suggestion and conditioning; however, for this analysis, only three studies were available, and only experimental pain was investigated [68].

In a recent study, placebo effects in experimental as well as chronic pain of patients with musculoskeletal pain did not differ when induced by verbal suggestion or a combination of verbal suggestion and conditioning. Yet, larger placebo responses in chronic pain were found in responders with more negative treatment history [74], indicating that prior experience plays an important moderating role, which, however, could be mediated by expectations. Investigating patients with atopic dermatitis with experimental electric pain, placebo hypoalgesia upon administration of a placebo cream was observed after verbal suggestion, after conditioning without verbal suggestion, and after a combination of both. Without conditioning, however, the effect quickly diminished, suggesting that conditioning compared to verbal suggestion leads to longer lasting placebo hypoalgesia in patients [40]. Finally, Klinger et al. [69] tested four different groups with chronic low back pain in clinical pain, in an experimental electric pain model, in self-rated functional capacity, and in a behavioral test using time needed to perform standardized daily activities. Patients received a sham opioid solution and were either instructed that they received a placebo (PI) or a pain-reducing opioid solution (OI). Furthermore, half of the participants underwent a conditioning procedure, resulting in two more groups (PI + Cond and OI + Cond). Opioid instruction led to large placebo effects on clinical and experimental pain, to decreased time needed for the exercises (small effect), and to increased self-rated functional capacity (medium effect). The combination with conditioning (OI + Cond) led to larger effects than OI on all outcome variables. The placebo-instructed group with conditioning showed a small yet significant effect for the time needed to perform exercises. These results support the notion that conditioning incrementally contributes to placebo hypoalgesia in patients with low back pain because the effects grew larger when a combination of conditioning and verbal suggestion was used, and the behavioral outcome measure showed placebo effects even in the absence of verbal suggestions.

The available studies indicate that both verbal suggestion and conditioning are powerful determinants of placebo hypoalgesia in clinical populations. However, not enough data have been gathered yet to identify the relative significance or an independent share of conditioning compared to explicit expectation in the clinical context. From a theoretical perspective, it can be assumed that conditioning constitutes an important aspect in chronic pain [75]. Especially, extinction has shown to be deficient in patients with chronic pain [76]. Furthermore, phenomena like latent inhibition (i.e., poorer learning as an effect of ineffective preexposure) or blocking effects (i.e., ineffective responding to a second conditioned stimulus) increase the risk of negative treatment experiences. Repeated experience of therapy failures, which is common in chronic pain, might lead to weakened placebo or persistent nocebo effects potentially contributing to the maintenance of chronic pain. However, due to ethical constraints, nocebo hyperalgesia cannot be investigated as rigorously as placebo hypoalgesia in clinical samples, and most knowledge in the realm is derived from studies on the disclosure of possible adverse effects in clinical trials [77]. On the other hand, there is limited evidence that conditioned placebo effects are longer lasting compared to effects that had been induced by explicit expectation [40, 78], and this could be an opportunity for the treatment of clinical pain conditions.

## 6. Conclusion and Future Directions

In sum, conditioning usually leads to the development of explicit expectation or (more technically) contingency awareness. Furthermore, it is not surprising that such an expectation is able to enhance placebo hypoalgesia and nocebo hyperalgesia as numerous studies have shown when comparing the effects after verbal suggestion and conditioning with verbal suggestion, respectively [27, 28]. However, this does not exclude the existence and impact of conditioned effects that do not depend on explicit expectation. Accordingly, Amanzio and Benedetti [34] elegantly showed that placebo hypoalgesia that had been conditioned with ketorolac and enhanced by a verbal suggestion was only partly reversed by the opioid antagonist naloxone, that is, naloxone only reversed the placebo effect induced by the expectation part, as the other experimental groups indicated. It is conceivable that conditioned effects can be compensated by or at least interact with contrary explicit expectations [10, 39]. This does not mean, however, that an expectation-independent, conditioned effect does not exist. We assert that there is not only one way to elicit placebo and nocebo effects in pain that is mediation by explicit expectations. Rather, classical conditioning on its own can generate these effects, which can be substantial in size, as available evidence shows for experimental as well as clinical pain.

One possibility to avoid confounding between induction by expectation and conditioning and to advance our understanding of the mechanisms causing placebo hypoalgesia and nocebo hyperalgesia would be the implementation of implicit conditioning designs. Here, an involvement of explicit expectations is excluded, and effects purely caused by conditioning can be investigated. Compared to conditioning with subliminally presented cues, implicit conditioning has some advantages, as discussed above. Another way would be the development of experimental designs, in which contrary placebo or nocebo effects are induced by conditioning and explicit expectation, and these effects are measured on independent variables.

## References

[1] Oken B. S. (2008). Placebo effects: clinical aspects and neurobiology. *Brain*.

[2] Stewart-Williams S., Podd J. (2004). The placebo effect: dissolving the expectancy versus conditioning debate. *Psychological Bulletin*.

[3] Colloca L., Lopiano L., Lanotte M., Benedetti F. (2004). Overt versus covert treatment for pain, anxiety, and Parkinson’s disease. *Lancet Neurology*.

[4] Benedetti F. (2002). How the doctor’s words affect the patient’s brain. *Evaluation & the Health Professions*.

[5] Benedetti F. (2008). Mechanisms of placebo and placebo-related effects across diseases and treatments. *Annual Review of Pharmacology and Toxicology*.

[6] Tracey I. (2010). Getting the pain you expect: mechanisms of placebo, nocebo and reappraisal effects in humans. *Nature Medicine*.

[7] Zhang W., Robertson J., Jones A. C., Dieppe P. A., Doherty M. (2008). The placebo effect and its determinants in osteoarthritis: meta-analysis of randomised controlled trials. *Annals of the Rheumatic Diseases*.

[8] Patel S. M., stason W. B., legedza A. (2005). The placebo effect in irritable bowel syndrome trials: a meta-analysis. *Neurogastroenterology and Motility*.

[9] Vase L., Vollert J., Finnerup N. B. (2015). Predictors of the placebo analgesia response in randomized controlled trials of chronic pain: a meta-analysis of the individual data from nine industrially sponsored trials. *Pain*.

[10] Benedetti F., Pollo A., Lopiano L., Lanotte M., Vighetti S., Rainero I. (2003). Conscious expectation and unconscious conditioning in analgesic, motor, and hormonal placebo/nocebo responses. *Journal of Neuroscience*.

[11] Voudouris N. J., Peck C. L., Coleman G. (1985). Conditioned placebo responses. *Journal of Personality and Social Psychology*.

[12] Voudouris N. J., Peck C. L., Coleman G. (1989). Conditioned response models of placebo phenomena: further support. *Pain*.

[13] Voudouris N. J., Peck C. L., Coleman G. (1990). The role of conditioning and verbal expectancy in the placebo response. *Pain*.

[14] Colloca L., Benedetti F. (2009). Placebo analgesia induced by social observational learning. *Pain*.

[15] Vögtle E., Barke A., Kröner-Herwig B. (2013). Nocebo hyperalgesia induced by social observational learning. *Pain*.

[16] Kirsch I. (1985). Response expectancy as a determinant of experience and behavior. *American Psychologist*.

[17] Price D. D., Milling L. S., Kirsch I., Duff A., Montgomery G. H., Nicholls S. S. (1999). An analysis of factors that contribute to the magnitude of placebo analgesia in an experimental paradigm. *Pain*.

[18] Wickramasekera I. (1980). A conditioned response model of the placebo effect. *Biofeedback and Self-Regulation*.

[19] Świder K., Bąbel P. (2013). The effect of the sex of a model on nocebo hyperalgesia induced by social observational learning. *Pain*.

[20] Hunter T., Siess F., Colloca L. (2014). Socially induced placebo analgesia: a comparison of a pre-recorded versus live face-to-face observation. *European Journal of Pain*.

[21] Egorova N., Park J., Orr S. P., Kirsch I., Gollub R. L., Kong J. (2015). Not seeing or feeling is still believing: conscious and non-conscious pain modulation after direct and observational learning. *Scientific Reports*.

[22] Colloca L., Miller F. G. (2011). How placebo responses are formed: a learning perspective. *Philosophical Transactions of the Royal Society B: Biological Sciences*.

[23] Bingel U., Wanigasekera V., Wiech K. (2011). The effect of treatment expectation on drug efficacy: imaging the analgesic benefit of the opioid remifentanil. *Science Translational Medicine*.

[24] Colloca L., Sigaudo M., Benedetti F. (2008). The role of learning in nocebo and placebo effects. *Pain*.

[25] Colloca L., Tinazzi M., Recchia S. (2008). Learning potentiates neurophysiological and behavioral placebo analgesic responses. *Pain*.

[26] Corsi N., Colloca L. (2017). Placebo and nocebo effects: the advantage of measuring expectations and psychological factors. *Frontiers in Psychology*.

[27] Vase L., Riley J. L., Price D. D. (2002). A comparison of placebo effects in clinical analgesic trials versus studies of placebo analgesia. *Pain*.

[28] Petersen G. L., Finnerup N. B., Colloca L. (2014). The magnitude of nocebo effects in pain: a meta-analysis. *Pain*.

[29] de Jong P. J., Baast R., Arntz A., Merckelbach H. (1996). The placebo effect in pain reduction: the influence of conditioning experiences and response expectancies. *International Journal of Behavioral Medicine*.

[30] Reicherts P., Gerdes A. B. M., Pauli P., Wieser M. J. (2016). Psychological placebo and nocebo effects on pain rely on expectation and previous experience. *Journal of Pain*.

[31] Pollo A., Vighetti S., Rainero I., Benedetti F. (2003). Placebo analgesia and the heart. *Pain*.

[32] van Laarhoven A. I., Vogelaar M. L., Wilder-Smith O. H. (2011). Induction of nocebo and placebo effects on itch and pain by verbal suggestions. *Pain*.

[33] Pazzaglia C., Testani E., Giordano R., Padua L., Valeriani M. (2016). Expectation to feel more pain disrupts the habituation of laser-pain rating and laser-evoked potential amplitudes. *Neuroscience*.

[34] Amanzio M., Benedetti F. (1999). Neuropharmacological dissection of placebo analgesia: expectation-activated opioid systems versus conditioning-activated specific subsystems. *Journal of Neuroscience*.

[35] Benedetti F., Amanzio M., Rosato R., Blanchard C. (2011). Nonopioid placebo analgesia is mediated by CB1 cannabinoid receptors. *Nature Medicine*.

[36] Carlino E., Torta D. M. E., Piedimonte A., Frisaldi E., Vighetti S., Benedetti F. (2015). Role of explicit verbal information in conditioned analgesia. *European Journal of Pain*.

[37] Babel P., Bajcar E. A., Adamczyk W. (2017). Classical conditioning without verbal suggestions elicits placebo analgesia and nocebo hyperalgesia. *PLoS One*.

[38] Kirsch I., Kong J., Sadler P. (2014). Expectancy and conditioning in placebo analgesia: separate or connected processes?. *Psychology of Consciousness: Theory, Research, and Practice*.

[39] Montgomery G. H., Kirsch I. (1997). Classical conditioning and the placebo effect. *Pain*.

[40] Klinger R., Soost S., Flor H., Worm M. (2007). Classical conditioning and expectancy in placebo hypoalgesia: a randomized controlled study in patients with atopic dermatitis and persons with healthy skin. *Pain*.

[41] De Pascalis V., Chiaradia C., Carotenuto E. (2002). The contribution of suggestibility and expectation to placebo analgesia phenomenon in an experimental setting. *Pain*.

[42] Watson A., El-Deredy W., Bentley D. E., Vogt B. A., Jones A. K. P. (2006). Categories of placebo response in the absence of site-specific expectation of analgesia. *Pain*.

[43] Martin-Pichora A. L., Mankovsky-Arnold T. D., Katz J. (2011). Implicit versus explicit associative learning and experimentally induced placebo hypoalgesia. *Journal of Pain Research*.

[44] Jensen K., Kirsch I., Odmalm S., Kaptchuk T. J., Ingvar M. (2015). Classical conditioning of analgesic and hyperalgesic pain responses without conscious awareness. *Proceedings of the National Academy of Sciences*.

[45] Kalish H. I. (1954). Strength of fear as a function of the number of acquisition and extinction trials. *Journal of Experimental Psychology*.

[46] Schmid J., Theysohn N., Ga F. (2013). Neural mechanisms mediating positive and negative treatment expectations in visceral pain: a functional magnetic resonance imaging study on placebo and nocebo effects in healthy volunteers. *Pain*.

[47] Colagiuri B., Quinn V. F., Colloca L. (2015). Nocebo hyperalgesia, partial reinforcement, and extinction. *Journal of Pain*.

[48] Charron J., Rainville P., Marchand S. (2006). Direct comparison of placebo effects on clinical and experimental pain. *Clinical Journal of Pain*.

[49] Jepma M., Wager T. D. (2015). Conceptual conditioning: mechanisms mediating conditioning effects on pain. *Psychological Science*.

[50] Schafer S. M., Colloca L., Wager T. D. (2015). Conditioned placebo analgesia persists when subjects know they are receiving a placebo. *Journal of Pain*.

[51] Kaptchuk T. J., Friedlander E., Kelley J. M. (2010). Placebos without deception: a randomized controlled trial in irritable bowel syndrome. *PLoS One*.

[52] Carvalho C., Caetano J. M., Cunha L., Rebouta P., Kaptchuk T. J., Kirsch I. (2016). Open-label placebo treatment in chronic low back pain: a randomized controlled trial. *Pain*.

[53] Charlesworth J. E. G., Petkovic G., Kelley J. M. (2017). Effects of placebos without deception compared with no treatment: a systematic review and meta-analysis. *Journal of Evidence-Based Medicine*.

[54] Wager T. D. (2005). Expectations and anxiety as mediators of placebo effects in pain. *Pain*.

[55] Herrnstein R. J. (1962). Placebo effect in the rat. *Science*.

[56] Hayashi T., Ohashi K., Tadokoro S. (1980). Conditioned drug effects to d-amphetamine- and morphine-induced motor acceleration in mice: experimental approach for placebo effect. *Japanese Journal of Pharmacology*.

[57] Nolan T. A., Price D. D., Caudle R. M., Murphy N. P., Neubert J. K. (2012). Placebo-induced analgesia in an operant pain model in rats. *Pain*.

[58] Bräscher A. K., Kleinböhl D., Hölzl R., Becker S. (2017). Differential classical conditioning of the nocebo effect: increasing heat-pain perception without verbal suggestions. *Frontiers in Psychology*.

[59] Egorova N., Park J., Kong J. (2017). In the face of pain: the choice of visual cues in pain conditioning matters. *European Journal of Pain*.

[60] Jensen K. B., Kaptchuk T. J., Kirsch I. (2012). Nonconscious activation of placebo and nocebo pain responses. *Proceedings of the National Academy of Sciences*.

[61] Jensen K. B., Kaptchuk T. J., Chen X. (2014). A neural mechanism for nonconscious activation of conditioned placebo and nocebo responses. *Cerebral Cortex*.

[62] Cowey A. (2004). The 30th Sir Frederick Bartlett Lecture: fact, artefact, and myth about blindsight. *Quarterly Journal of Experimental Psychology Section A*.

[63] Becker S., Kleinböhl D., Hölzl R. (2012). Awareness is awareness is awareness? Decomposing different aspects of awareness and their role in operant learning of pain sensitivity. *Consciousness and Cognition*.

[64] Hölzl R., Kleinböhl D., Huse E. (2005). Implicit operant learning of pain sensitization. *Pain*.

[65] Goubert L., Crombez G., Hermans D., Vanderstraeten G. (2003). Implicit attitude towards pictures of back-stressing activities in pain-free subjects and patients with low back pain: an affective priming study. *European Journal of Pain*.

[66] Grumm M., Erbe K., von Collani G., Nestler S. (2008). Automatic processing of pain: the change of implicit pain associations after psychotherapy. *Behaviour Research and Therapy*.

[67] Haug M. (2011). Explaining the placebo effect: aliefs, beliefs, and conditioning. *Philosophical Psychology*.

[68] Peerdeman K. J., van Laarhoven A. I. M., Keij S. M. (2016). Relieving patients’ pain with expectation interventions: a meta-analysis. *Pain*.

[69] Klinger R., Kothe R., Schmitz J., Kamping S., Flor H. (2017). Placebo effects of a sham opioid solution: a randomized controlled study in patients with chronic low back pain. *Pain*.

[70] Vase L., Robinson M. E., Verne N. G., Price D. D. (2003). The contributions of suggestion, desire, and expectation to placebo effects in irritable bowel syndrome patients. An empirical investigation. *Pain*.

[71] Vase L., Robinson M. E., Verne N. G., Price D. D. (2005). Increased placebo analgesia over time in irritable bowel syndrome (IBS) patients is associated with desire and expectation but not endogenous opioid mechanisms. *Pain*.

[72] Lee H. F., Hsieh J.-C., Lu C.-L. (2012). Enhanced affect/cognition-related brain responses during visceral placebo analgesia in irritable bowel syndrome patients. *Pain*.

[73] Hashmi J. A., Kong J., Spaeth R., Khan S., Kaptchuk T. J., Gollub R. L. (2014). Functional network architecture predicts psychologically mediated analgesia related to treatment in chronic knee pain patients. *Journal of Neuroscience*.

[74] Muller M., Kamping S., Benrath J. (2016). Treatment history and placebo responses to experimental and clinical pain in chronic pain patients. *European Journal of Pain*.

[75] Vlaeyen J. W., Linton S. J. (2000). Fear-avoidance and its consequences in chronic musculoskeletal pain: a state of the art. *Pain*.

[76] Flor H., Knost B., Birbaumer N. (2002). The role of operant conditioning in chronic pain: an experimental investigation. *Pain*.

[77] Colloca L., Miller F. G. (2011). The nocebo effect and its relevance for clinical practice. *Psychosomatic Medicine*.

[78] Colloca L., Petrovic P., Wager T. D., Ingvar M., Benedetti F. (2010). How the number of learning trials affects placebo and nocebo responses. *Pain*.

